# Exploring the Impact of the COVID-19 Pandemic on Italy’s School-in-Hospital (SiHo) Services: The Teachers’ Perspective

**DOI:** 10.5334/cie.26

**Published:** 2020-12-11

**Authors:** Vincenza Benigno, Francesca Dagnino, Chiara Fante

**Affiliations:** 1 CNR, IT

**Keywords:** COVID-19, School-in-Hospital, distance learning

## Abstract

The abrupt interruption of face-to-face educational activities imposed by the Italian government in response to the COVID-19 pandemic required a rapid switch to distance learning to guarantee continuity in education. The same applied to the School-in-Hospital (SiHo) services. This paper explores the impact of the COVID-19 pandemic in this specific context; we investigated the organizational changes and strategies adopted by the SiHo services and individual teachers to meet the challenges posed by this change in the educational paradigm. Moreover, the paper outlines teachers’ visions about possible educational scenarios for the next academic year (2020–2021). Using qualitative methodology, the study took the form of a group interview involving 12 teachers working across 8 children’s hospitals in Italy. The thematic analysis revealed a number of themes for further investigation. In terms of the actual situation during the pandemic, we identified critical issues and facilitating factors that occurred in distance learning with hospitalized students. One significant theme that emerged from the interviewees’ narratives involved their general attitudes towards distance learning and their individual approaches to the pandemic. The relationship of hospitalized students to mainstream schools also proved to be an important theme, not only regarding the recent past but also the future. Finally, teachers hypothesized future distance learning scenarios for the new school year, highlighting the challenges to be faced from several viewpoints (methodological, organizational, technological, etc.).

## Introduction

The COVID-19 pandemic has had a disruptive effect worldwide on several fronts; not only has it put the health and economy sectors under severe strain, it has also caused significant upheaval in education (Bozkurt et al., 2020; Pourdehnad et al., 2020). In an effort to contain the spread of the virus, several countries opted for a general lockdown that included workplaces, schools, and people’s social lives in general. In Italy, through an administrative order called the Decree of the President of the Council of Ministers (DPCM) on March 9, 2020, all schools were closed, and distance learning was adopted instead.

As in many other countries, Italian teachers have had to face the challenge of moving from face-to-face (F2F) instruction to online activities; this required not only relevant technological skills, but also a rethinking of teaching methodologies (UNESCO, 2020b, Lurvnik). Many families, in turn, have struggled with limited availability of suitable digital devices and a lack of adequate Internet connections to accommodate distance learning from home. Further, the role of supporting their children’s online learning has been difficult for many parents, and last but not least, scarce attention has been paid to students with special needs (UNESCO, 2020a). Taken together, these realities have made ensuring continuity of teaching challenging, even in the most advanced countries where conditions are mostly favourable.

While the situation in mainstream education has been difficult, it has been even more problematic for students suffering from severe and/or chronic illnesses, especially if they have had to face frequent, long-term or periodical hospitalization. In Italy, the School-in-Hospital (SiHo) service guarantees education for these students. The COVID-19 pandemic has led to severe interruptions to their regular learning activities and has affected the traditional F2F relationship between the SiHo teachers and their students, requiring them to find rapid and effective solutions (Mantegazza, 2020). This paper explores the teachers’ perspective during this pandemic.

### The School-in-Hospital Services

Across the world, hospital schools have come into being in one form or another, and over the last few decades, many countries have passed specific laws to regulate their organization, responsibilities, and resources.

SiHo forms part of the universal guarantee to the right to an education (Art. 26 of the United Nations International Human Rights Declaration (United Nations, 1948) and offers hospitalized students a social context and a place of learning where they can continue to express themselves and develop.

The SiHo organization, which operates in all of Italy’s hospital paediatric departments, is the product of a lengthy process that led to the development of a European Charter of Rights for Children in Hospital (European Association for Children in Hospital [EACH], 2002). Article 7 of the charter reads: “Children shall have full opportunity for play, recreation and education suited to their age and condition … [conducted] in an environment designed, furnished, staffed and equipped to meet their needs; in particular [allowance shall be made] for the continuation of the level of education already reached.”

In recent years, SiHo has evolved in terms of its regulatory and organizational-pedagogical aspects. In Italy, SiHo is managed by the Centre School, which acts as a reference school in each region responsible for the coordination and preparation of teachers (who arrive without any specific training) and for the formal interactions between the hospital and the student’s mainstream schools. These responsibilities are stipulated in a special agreement that is signed and ratified by the local health authority.

A series of SiHo guidelines have been issued, including all the organizational-didactic aspects (Ministero dell’Istruzione, dell’Università e della Ricerca [MIUR], 2019). From a praxis viewpoint, teachers have developed several daily routines described following a comprehensive survey (Benigno et al., 2017) of 95% of hospital teachers in Italy.

Formally, the management of the SiHo pathway involves the following phases:

- Establishing how many children are present each day in the hospital and/or are in a physical condition to be able to take part in learning activities
- Introducing the hospital school to newly admitted young inpatients and their families
- Establishing contact with the newly hospitalized student’s mainstream school teachers to acquire information on his or her skill levels
- Evaluating the type of hospitalization; short stay (up to seven days), average stay (from 7 to 15 days) and long stay (over 15 days)
- Designing and implementing a learning pathway for the student
- Sending the student’s portfolio containing his or her work and results during time in hospital to the mainstream school at the end of hospitalization

The involvement of the family is the first and most important step in the process. Indeed, several authors have outlined the importance of parental support to mediate the work of the teacher (Benigno et al., 2017; Dixon, 2014; Hopkins et al., 2014; St Leger, 2014).

In addition, the SiHo context demands that the hospital teacher works closely with the hospital’s medical staff (Capurso & Dennis, 2017) to ensure both successful planning and implementation of instruction and, more generally, foster a positive and collaborative SiHo climate. The relationship between SiHo teachers and medical staff is informal; information on specific students is exchanged when deemed necessary, and only in some hospital contexts is the teacher expected to attend healthcare team meetings (Benigno et al., 2017).

Hospital teachers organize their teaching activities around the students’ state of physical health and psychological wellbeing, as well as needs triggered by the experience of illness and isolation (Shaw & Brown, 2011). Furthermore, learning pathways have to be customized according to the heterogeneity of the students, particularly in relation to age, skill level, and length of hospital stay. Given the complexity of this educational scenario, teachers often opt for an individualized approach (which changes from student to student) and, where possible, conduct some activities in small groups (Benigno et al., 2017).

Communication and collaboration with the mainstream school is mostly limited to the exchange of formal documents, as reported by Benigno and colleagues (2017). However, contact between the hospital and mainstream schools is beneficial for other aspects connected to the emotional wellbeing of the hospitalized students and is necessary for educational continuity.

The hospital teacher’s role should operate within a multidisciplinary context and as part of a holistic approach (Brofenbrenner, 1994) to encourage remote interactions among teachers and to create a sense of continuity with the student’s life prior to hospitalization. Some authors (e.g., Hopkins et al., 2014; St Leger, 2014) see the principal mission of SiHo as a reimagining and redefining of the hospital environment as a multidisciplinary space dedicated to individual health, development, wellbeing and learning – a role that goes well beyond the provision of traditional medical care.

During the current COVID-19 pandemic, hospital teachers have had to deal with increasingly complex problems linked to their presence in the workplace (hospitals), such as performing their role as educators in that setting given restrictions due to COVID-19.

### The Present Study

This study analysed the impact of the COVID-19 pandemic on the SiHo services in Italy. It was carried out within the framework of the CLIPSO project (hybrid classes for SiHo; https://www.progetto-clipso.it/), which aims to overcome the isolation of hospitalized children and to support their inclusion in the curricular activities of their mainstream schools. This is achieved through the appropriate use of educational technologies and the promotion of collaboration between the SiHo services and the mainstream (home) schools.

Impact analysis was performed by investigating the organizational changes and strategies adopted by the SiHo services and single teachers in response to the abrupt interruption of F2F activities and the subsequent switch to distance education. Moreover, the study sought to collect teachers’ opinions about possible educational scenarios for the 2020–21 school year. The study was carried out using qualitative methodology, specifically a group interview (Brenner, 2006) with 12 teachers working in 8 children’s hospitals or wards in Italy.

The research questions (RQs) were as follows:

RQ1: How have the SiHo services been managed during the COVID-19 pandemic to ensure educational continuity for hospitalized students?

RQ2: What methodological and organizational aspects should be considered if distance learning becomes a universal requirement for mainstream educational activities in Italy during the 2020–2021 school year?

## Methods

### Participants

The study involved 12 teachers working in 8 children’s hospitals or wards in Italy; 4 were located in northern Italy, 1 in central Italy, and 3 in southern Italy. The teachers were all female, and their teaching covered all school levels (some taught at more than one level); two kindergarten, three primary, six lower secondary, and three upper secondary. Participants’ SiHo experience ranged from 1 year of teaching to over 20 years’ experience.

Participating teachers were recruited in April 2020 via an email invitation sent to a group of SiHo teachers who had registered on a project platform dedicated to reporting on teachers’ SiHo experiences and sharing materials during the COVID pandemic.

### Procedure

The research team formulated two overarching questions that were presented to the subjects in a semi-structured group interview conducted online by members of the research team. The two questions were discussed over two separate interview sessions; the first was carried out during the last week of April 2020 (Question 1), and the second question was discussed two weeks later, in May 2020 (Question 2). The two-session format was agreed upon with the participants after the first session, as the discussion of Question 1 turned out to be quite protracted. Both interview sessions were video-recorded and subsequently transcribed verbatim by a member of the research team. Before any interviewing activities started, participants gave informed consent to the recording, processing, and use of the material collected.

The first interview session started with a brief overview of the effects of the decision made by the Ministry of Education in relation to the COVID pandemic (i.e., the sudden suspension of all F2F educational activities), which not only prevented SiHo teachers from meeting with their students but also stopped them from entering the hospitals where they worked. This restriction posed several challenges to maintaining the SiHo service, and led to the first question, as follows:

1. How did you manage to guarantee continuity of service during the COVID health pandemic?

During the first interview session, the interviewer’s questions encouraged teachers to describe the limitations they faced, strategies adopted, and the outcomes of their efforts.

The second interview session in May focused on the future, specifically on possible educational scenarios for the beginning of the next school year if resumption of F2F lessons would not be possible. The question was as follows:

2. If the current conditions were to remain in place at the beginning of the 2020–2021 school year, and you have to design distance activities for your SiHo students, what aspects would you consider at the organizational and methodological levels, and what tools would you use?

In this second session, the interviewers prompted teachers to explore the situation, not just from an organizational level (e.g., how to make contact with the students), but also from a methodological viewpoint.

Attention was also paid to the relationship with the mainstream schools of the children that, as became clear from the first part of the interview, have been in charge of including hospitalized children in their online activities during the COVID.

#### Analysis of the Interviews

Given the descriptive and explorative nature of the research goals, analysis of the interview data was carried out using a thematic analysis approach that aimed to identify and analyze patterns (or themes) within a set of data (Braun & Clarke, 2006; Kelle, 2007). Themes are developed through the analysis process rather than being defined beforehand. A theme is a cluster of codes conveying similar meanings, whereas a code is the primary product of the analytical process at the most fundamental descriptive level (Vaismoradi et al., 2016). The analysis was carried out in an inductive or bottom-up manner; that is, the researchers were not guided in any way by preconceptions or hypotheses, so the process was fully inductive. Moreover, the analysis was semantic, and themes were identified within the explicit meanings of the data (Braun & Clarke, 2006).

The coders (three psychologists who conducted the interviews) familiarized themselves with the interview transcriptions at the start of the analysis process through multiple readings. Next, they separately coded the texts and generated individual code sets for both interviews. Subsequently, they compared their code category sets to develop a single framework. Interpretive differences were resolved through discussion and negotiation. Finally, a “code book” (a set of codes and related definitions) was established that included the codes from both interviews. This ensured that coding coherence and consistency was achieved through a two-step process – individual parallel coding and subsequent negotiation of differences in the generated codes. Subsequently, the coders re-read the interview transcripts using the joint codebook to code any specific portions of text that each deemed attributable to the newly identified codes. At the end of the coding process, a joint review was conducted to resolve any discrepancies that emerged during the individual coding phase.

The individual coding phase using the codebook was carried out using MAXQDA, a program specifically designed for qualitative analysis. The program not only allows codebook-guided coding of texts but also enables analysis and reporting of results in graphic and textual forms (De Gregorio & Lattanzi, 2011; Kuckartz & Rädiker, 2019).

## Results

Table 1 (see Appendix) lists the main issues that emerged from the two-part interview process and the related themes and codes that resulted from the qualitative analyses. The results are primarily presented and discussed in terms of the responses to the two research questions. One main theme emerged from the analysis; that is, the general attitude of teachers towards distance learning and their reaction to the pandemic phase (Themes: “Teachers’ attitudes towards distance learning and the pandemic”).

**Figure 1:**
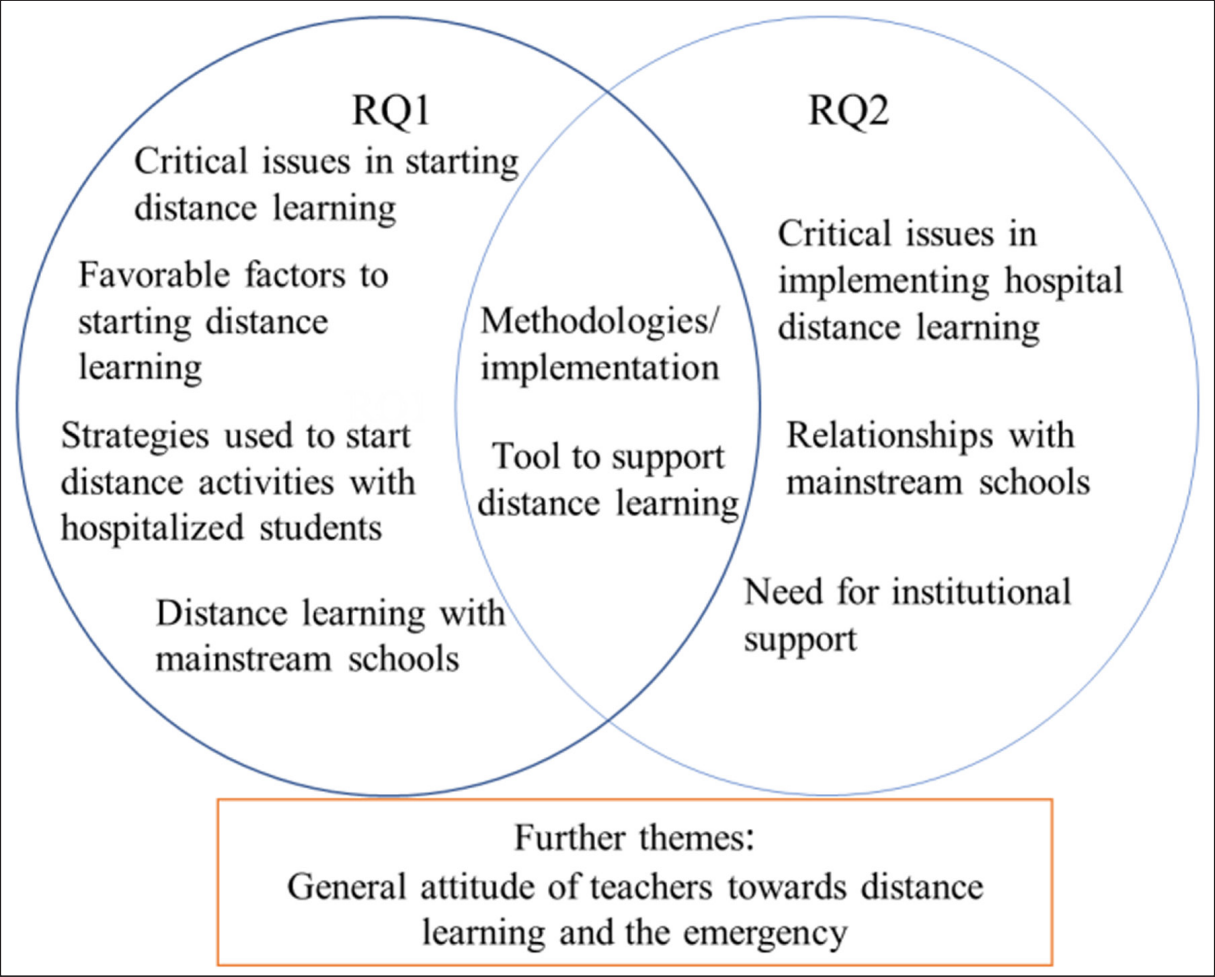
Organization of Themes in Relation to the RQs.

Organization of the themes in relation to the two research questions is presented in Figure 1.

### RQ1: How have the SiHo services been managed during the COVID-19 pandemic to ensure educational continuity for hospitalized students?

In relation to the organization of hospital teaching during the first phase of the health pandemic, the main themes that were identified concerned (a) critical issues in starting distance learning; (b) factors favorable to starting distance learning; (c) strategies used to start distance activities with hospitalized students; (d) tools to support distance learning; (e) methodologies and implementation strategies; and (f) distance learning with mainstream schools.

#### Critical Issues in Starting Distance Learning

Teachers reported organizational changes in hospital wards following the health pandemic; limitations in routine hospital activities and an overall reduction in hospitalizations had a negative impact on the continuity of SiHo services.

The inability to physically access hospital spaces caused considerable difficulties in establishing contact with new students and their families, and led to an overall reduction in SiHo activities.

*From the very beginning we had the problem of … not being allowed to enter the wards, the hospital school, and not being allowed to access our documents…Up until last year the digital school register was accessible to us online, but this year the Ministry no longer allowed access so we had to use manual registers, (so) all our data were inaccessible in the hospital, and so was a bit of an issue initially*.

A further obstacle to initiating contact was the teachers’ inability to access the daily list of students admitted to the wards; in addition, contacting newly hospitalized young patients was also hindered by aspects related to privacy.

*… Well, we didn’t have any new [student] applications, so, for next year we will need to find a way to obtain reports of those hospitalized students. In previous years we used to get a report from the hospital, but we have been informed that we can no longer have this due to privacy issues. However, we will have to solve this issue for the next [school] year because either they will have to let us enter the hospital and collect the new applications, or we will need a report provided by the hospital; otherwise, we won’t be able to do our job*.

In addition, respondents reported encountering difficulties in initiating or maintaining school services with short-term patients due to a lack of time caused by not being able to contact new students quickly.

A further critical element that inhibited implementation of distance learning was the relationship with healthcare staff; respondents reported poor communication with nurses and doctors, and non-recognition of the teacher’s role as factors that prevented them from doing their jobs.

*Unfortunately, the hospital school teacher, in other wards, is often considered as… I don’t want to say as an interloper, but as an optional extra, who…, well … whose presence doesn’t make much difference either way*.

#### Factors Favorable to Starting Distance Learning

Initiation of hospital contacts and remote teaching was facilitated by variables such as the existence of pre-pandemic relationships with students and/or their families, the length of hospital stays, and the students’ age; maintaining remote learning activities was easier with older students and those with lengthy hospital stays.

*I teach high school students so they’re older, and most of them attend grammar school or a technical school of some sort, so they are motivated not to lose time … that made it easier to resume working under the current difficult circumstances. What I’ve experienced is continuity; they were all hospitalized or attended the day hospital, and have all been receiving treatment for several months*.

Overall, from the teachers’ perspective, the affective/relational dimension was a determining factor; they were able to continue their activities more easily if they were able to collaborate with healthcare personnel and with the students’ families. The families were a particularly important source of support; they responded positively to the service and formed a solid collaboration. The teachers were able to reorganize their activities during the early stages of the pandemic due to their ongoing relationships with their hospital school colleagues and their counterparts at other hospital schools.

*How did we manage to proceed? Basically, we relied on teamwork involving teachers working at different levels. So we maintained our weekly meetings between teachers from the different levels (primary, lower, and upper secondary), even though our work was in different schools. We also continued our weekly meetings with healthcare staff, and this allowed us to develop our strategy to reach students and parents*.

Finally, the professional/organizational flexibility usually required of hospital teachers helped them to cope with organizational changes and in restarting the service.

#### Strategies Used to Start Distance Activities With Hospitalized Students

The teachers adopted several strategies in an effort to initiate distance learning activities, but in some cases, they turned out to be ineffective. For example, they promoted their activities by emailing posters to healthcare personnel to display in departments to “advertise” the SiHo service and to spread the word among their contacts. However, in most cases, this failed to increase contact with the students and their families. The service was also promoted via the hospital’s social media channels, but this approach also proved to be largely ineffective. Generally, the actions that were effective consisted either of direct telephone contact with students already enrolled and their families or direct contact with hospital staff to obtain the contact data.

*We managed to get our student list for both mid- and long-term patients, and we contacted them directly by speaking with the parents and asking whether they needed our involvement to keep their child in touch with their educational activities*.

The interviewees also reported that they collaborated with colleagues to identify the technology they would use and to discuss privacy issues. They engaged (voluntarily) in distance learning training to further their professional development to help face the challenges presented by the pandemic.

#### Tools to Support Distance Learning, Methodologies, and Implementation Strategies

Although most teaching activities have been reduced dramatically, some teachers reported that they were able to activate distance learning with hospitalized students. In those cases, the teachers conducted video lessons, often using tools already familiar to the students (e.g., Skype and Google Meet). During these remote activities, teachers adopted 1:1 teaching, individualized lessons, and short teaching sessions or created small workgroups involving students who were hospitalized on different wards. Moreover, some students asked to do some remedial or catch-up studies with their mainstream school. The availability of hospital school teachers to personalize the educational activities was an essential benefit for students who were not able to take part in the distance activities run by their mainstream school.

*… those we are teaching are either severely ill or are not in a condition to follow online lessons with their classmates, or do not fully understand their regular class lessons … therefore, we teach [with the aim] of strengthening their knowledge and competences*.

The interviewees also mentioned other possible solutions to assist with distance education, such as implementation of an “educational helpdesk” and setting up learning content repositories.

#### Distance Learning With Mainstream Schools

During the first part of the interview, teachers stated that in many cases hospitalized students were able to follow the distance learning programs implemented by their mainstream schools and that joining activities with their classmates was easy. For some students, the hospital teacher played an important role in supporting the distance activities with their schools and in mediating their return to mainstream-school distance learning.

Although hospitalized students showed initial enthusiasm for attending remote activities with their peers, some critical issues emerged. In particular, distance learning was difficult in cases where the students had poor relationships with their peers and/or suffered from critical health conditions. It was also difficult for students from immigrant backgrounds whose parents had language difficulties, which, in turn, affected their use of the technology. In addition, the distance education run by the mainstream schools did not consider the different times dedicated to learning activities during the hospital stay and generated an excessive workload.

*A father described his child’s initial enthusiasm to me and how his son followed the daily mainstream (home) school live lessons almost doggedly. But this interest steadily waned, and in the end, he was finding the whole experience emotionally trying, somewhat overwhelming, and draining. I got a different impression; actually, the child had literally thrown himself into this virtual classroom, finding himself amongst a group of students that he really didn’t know since he’d been hospitalized at the beginning of September. So he hadn’t had the chance to get to know his classmates*.

### RQ2: What methodological and organizational aspects should be considered if distance learning becomes a universal requirement for mainstream educational activities in Italy during the 2020–2021 school year?

The main themes identified in the second interview relating to RQ concerned (a) tools to support distance learning; (b) methodologies and implementation strategies; (c) critical issues in implementing hospital distance learning; (d) relationships with mainstream schools; and (d) the need for institutional support.

#### Tools to Support Distance Learning, Methodologies, and Implementation Strategies

If distance learning has to continue during the initial months of the 2020–21 school year, respondents considered the methods already tried during the pandemic, such as video lessons and synchronous activities, potentially effective options. Teachers acknowledged the need to be flexible when selecting technological tools and were aware that they should prioritize tools that students already use. However, at the same time, they were less aware of how this can facilitate continuity in the students’ learning paths.

Teachers recognized the complexity of the online methodological approach, especially in terms of changing from a purely transmissive methodology to include asynchronous activities. Some respondents viewed planning activities with students and mixing courses with mainstream schools as useful.

*It is a strictly methodological-didactic matter, and so calls into question our roles as teachers … at least as far as I am concerned, because up until now we have attempted to adapt our normal teaching practice and, as someone said before … maybe F…, this is not actually … I’m aware this isn’t actually real distance education; far from it. We’re teaching [online] but we should engage with this methodology at a deeper level*.

In this regard, the teachers cited the need for suitable training.

#### Critical Issues in Implementing Hospital Distance Learning

Our analysis of the interviews shed light on several critical issues in hospital distance learning. One of the main factors was initiating distance contacts, and this may well remain a critical issue in the future. The inability to access hospital spaces and to have F2F contact with families and students were also limitations to teaching.

*Well, in my view, direct personal contact can have positive effects. First of all, meeting up and actually seeing each other has more impact than a phone call. If you get the chance, and have a phone call with a parent, then you can actually start your distance teaching. But this is essential … meeting the learner’s relatives in person*.

Another critical area was the use of technological devices and tools. Some teachers felt these were ineffective during distance activities, or they encountered students and families with low technological skill levels. In addition, younger students needed parental support to use the technology and to manage their remote lessons.

One of the most critical issues stressed by teachers was the establishment of good relationships with the students and their families, especially when the students were from an immigrant background.

*So, in the first phase …. [of the pandemic lockdown] …. getting used to distance learning doesn’t help the students much. We are here to look at new approaches; a new way to establish relationships with these children, who, before setting out on their individual learning paths, need to connect with their teachers, form a relationship with a person they can trust, someone with whom they can start on a new journey, even though it might be a hard one, but nonetheless, a journey during which they will develop a strong sense of empathy, and educational companionship*.

Another critical aspect that emerged concerned the teachers’ workload (code “Effort in carrying out distance learning”).

#### Relationships With Mainstream Schools

Another code that emerged from the analysis concerned the possible benefits of hospital school distance learning activities. Students undergoing long-term hospitalization could benefit more from the service, while those who were only hospitalized for a short period could follow the remote activities implemented by their schools.

*Actually, in my experience, that’s not the case. I mean, children who are ill have particular frailties and needs that often, for reasons we can’t go into now, don’t allow them to cope with the dynamics of distance learning conceived for a [whole] class. In that environment, [teachers] can’t find room for them for a variety of reasons, such as these students are not aligned with school programs, because the workload is too heavy, or because synchronous lessons are carried out at times that are incompatible with their treatments*.

Distance learning with hospitalized students requires rapid and active collaboration with their mainstream schools and with classmates to develop suitable learning units. Teachers recognize the importance of creating collaborative working conditions and allowing students to maintain social relationships with their peers.

Respondents mentioned the creating of a single remote class that would include all the students (i.e., those who are hospitalized and their classmates) as a possible future solution for emergency education.

*… Why don’t we consider extending the class, where … maybe we are there together with our hospitalized students and their school class … maybe we could offer our support or make suggestions or help or individualize or … “personalize” their learning*.

#### Need for Institutional Support

To be effective, remote hospital teaching requires collaboration between different professional representatives and organizational strategies. At the institutional level, teachers wanted the publication of national guidelines with which they can align their teaching activities and from which they can gain practical recommendations about student management. They also emphasized needing the support of school principals during the organizational and planning phases.

In a hospital context, teacher involvement as part of the healthcare team is deemed necessary to support teaching activities; however, respondents were aware that the role of the hospital teacher has yet to be recognized by healthcare professionals. The teachers advocated for the appointment of a contact person within the healthcare team to enable effective collaboration during the child’s health crisis.

*… so if school is considered therapeutic in a children’s hospital, it should be part of the treatment program …… only if the teacher becomes a link in the chain will we be notified of the students’ presence*.

Respondents felt that promoting the hospital school and disseminating information about the service in different contexts could facilitate maintenance of teaching activities during the COVID-19 pandemic.

#### Teachers’ Attitudes Towards Distance Learning and the Pandemic

The first phase of the pandemic and the consequent lockdown (March 2020) aroused different feelings and reactions in the teachers. Some interviewees reported initial disorientation related to changes in work routines and defined the first weeks of the pandemic as critical. However, other teachers perceived the change as a new professional experience.

Overall, the interviewees showed a generally positive attitude towards distance learning, as well as an openness both towards the students and their families and towards the adoption of new professional practices. However, there was also some resistance to the adoption of remote teaching solutions.

Listening to the experience of colleagues from other hospitals sparked feelings of gratitude and shed light on similarities and differences in their respective work environments. From the teachers’ viewpoints, the health pandemic required a general redefinition of their work; however, the desire to return to “normality” and to their own routines was also highlighted.

*I mean, we keep working, and later on down the track I think things will gradually change and, well, the patient population will become a bit more, maybe not “normal,” but more numerous than now*.

## Discussion

In terms of the first research question investigating how teachers kept the hospital school going during the COVID health emergency phase, a number of issues emerged. Some of these are completely new, while others already existed, confirming the complexity of the hospital teacher’s work context (Benigno et al., 2017).

During the lockdown, just like traditional school teachers, hospital teachers suddenly had to switch from F2F to distance teaching (UNESCO, 2020b, Lurvnik). Considering that in standard hospital schooling, teachers work directly with their students in their hospital rooms and wards (Benigno et al., 2017), maintaining contact with students and their families would have necessitated the mediation of an “education/healthcare mediator” figure.

However, the lack of formal communication and coordination with healthcare professionals in many cases led to an interruption of hospital school activities, reinforcing the feeling of professional non-recognition that many hospital teachers already felt. Thus, the COVID pandemic seems to have intensified the difficulties they usually experience with interpersonal relationships, and this stressor has been identified by hospital teachers elsewhere (Benigno & Fante, 2020).

Informal contact with health professionals and direct contact with families were perceived as the most effective strategies to keep in touch with students, while other actions, such as using promotional posters and notices in hospital departments, were ineffective due to the lack of human mediation.

Our results highlight the need to build a network of interdisciplinary professionals to integrate the teacher more fully within the hospital context. Furthermore, these findings indicate that starting learning in a hospital means that teachers have to build trusting relationships with all the various parties involved.

During the initial phase of the pandemic, several teachers were able to start remote teaching, albeit with only a few students. For distance learning, the teachers mainly used tools supporting video lessons and other applications with which their students were already familiar. Furthermore, the relationship was mainly 1:1, and they rarely performed any type of small group activities. The educational activity was mainly aimed at supporting students in aligning their studies with their mainstream class programs. The management of distance teaching activities seems to largely reflect the standard methods that hospital teachers usually adopt. The lack of educational innovation may be due to the sudden demand to deal with a very critical situation and respond to the new working context as best they could.

During the emergency phase, the relationship between hospitalized students and their mainstream classes, which normally tends to be limited (Dixon, 2014), was intensified. Many students initially participated with enthusiasm in the distance activities that their mainstream school implemented. However, this first “scramble” phase has not been followed by carefully planned and effective teaching interventions. Additionally, the distance learning initiatives implemented by many mainstream schools did not consider the particular educational needs of hospitalized students. These needs concern the particular health conditions of the students and require the adaptation of programs, objectives, and strategies employed. Education for hospitalized students must also take into account specific organizational aspects, such as making allowance for treatment times that may prevent hospitalized learners from following lessons held during normal school hours.

Our findings confirm how unprepared most mainstream schools to address the educational and psychological needs of students who are ill (Mukherjee et al., 2000). We also found that closer communication between hospital teachers and teachers in mainstream schools is essential to establish suitable teaching interventions, a practice that could also support students’ return to their mainstream school (Hopkins et al., 2014). In this regard, it must be kept in mind that returning to school after hospitalization or a long absences due to illness represents a critical moment for young students, one that is connected to the risk of school dropout and the development of psychopathologies and emotional disorders (Hopkins et al., 2014; Martinez & Ercikan, 2009).

The initial phases of the pandemic emergency left many hospital teachers feeling disoriented and stimulated them to share their experiences and the need for mutual support. Considering the unpredictable nature of the pandemic, teachers had to plan flexible and alternative educational scenarios for the coming school year. This was the focus of our second research question. The teachers interviewed were generally positive about the adoption of distance learning when they were not able to access the hospital premises. This openness was supported by an awareness that effective distance learning requires a range of technological and methodological competences.

At the same time, however, teachers reported several critical issues. First, they reported difficulty in initiating contact with students and families without either having the active collaboration of healthcare personnel or being able to implement new strategies to collect applications (such as the lists of newly hospitalized students). According to the interviewees, distance also hinders the creation or maintenance of meaningful relationships with students, which is essential to carrying out any type of meaningful educational activities. This reflects limited awareness of the affective/emotional aspects of distance learning (Juutinen & Saariluoma, 2010).

Another perceived weakness was the low-level technological competency of some hospital teachers, but also of some families (whose support is vital) and some students. Previous studies have investigated teachers’ technological competencies (Vongkulluksn et al., 2018), as well as students’ digital competencies (Hatlevik et al., 2015). However, family competencies in the use of technology requires further investigation.

In terms of extending distance learning, the participants highlighted the importance of collaboration with mainstream schools. A collaborative relationship would allow hospital teachers to perform a support role in mediating between the students and schools, especially for long-term patients who, as stated earlier, experience problems accessing the distance educational activities of their mainstream and need personalized learning. Hospital teachers could play a crucial role in supporting activities implemented by mainstream schools, both in the presence and absence of future emergency conditions. This close relationship is considered paramount to fostering a sense of inclusion for hospitalized students with their classmates.

Another aspect that emerged from our study concerned the institutional dimension; teachers lamented the lack of national guidelines (the Italian Ministry of Education did not provide specific indications for the SiHo services during the pandemic) and the lack of active involvement of school principals.

## Conclusion

This study explored the impact of the COVID-19 pandemic on the SiHo services. Analysis of teacher interviews highlighted two general types of issues: (a) issues that can be considered as pre-existing factors affecting this specific context before the lockdown and that were exacerbated by the pandemic; and (b) issues that resulted from the specific situation.

One of the pre-existing issues was the inclusion of hospitalized children in mainstream school activities. In standard SiHo activities, this inclusion is usually very limited. The pandemic and the related switch to distance education presented the opportunity for hospitalized students to participate in mainstream online school activities, but where this took place, it was done without mainstream teachers or schools being fully aware of the special needs of these students. This finding supports the notion that specific interventions designed to foster the inclusion of these students through the use of technology should be proposed to teachers both in mainstream and hospital schools so that learning experiences are respectful of the specific needs of the hospitalized students. This is the mission of the CLIPSO project, which has a remit to support hospitalized students’ social-educational inclusion and to develop guidelines for school re-entry.

The hospital teachers interviewed also appeared to have limited awareness of the emotional aspects involved in distance learning. This is further evidence for the potential usefulness of specific training designed to increase teachers’ competency in establishing distance educational relationships. Another issue was the digital competency of the different people involved (i.e., teachers, students, and families). In particular, the digital competency of students’ families requires further research. This is especially vital because during the lockdown, parents provided essential support for their children’s distance learning, especially in the hospital context.

Finally, at the institutional level, the pandemic has highlighted some pre-existing critical issues within the profession that are connected to a lack of professional recognition by and involvement with the healthcare team (Benigno & Fante, 2020; Benigno et al., 2017). This finding highlights the need to rethink the formal role of teachers within the hospital context. Moreover, considering the educational needs of hospitalized students, as emerged from the interviews, the teachers in the hospital could act as catalysts to launch and manage new inclusive hybrid spaces with the school classes. The feelings of isolation and disorientation felt in recent months by the teachers interviewed translate into a clear need for greater collegiality and the desire to build a community that is capable of supporting the sharing of experiences and ideas (McArdle & Coutts, 2010).

## Limitations of the Study

This study has some limits, primarily regarding the recruitment of participants. The interviewees were recruited from a teacher cohort that had already registered on an online platform dedicated to supporting distance learning for SiHo services. For this reason, the sample cannot be considered as random or representative. Moreover, the study was dedicated solely to the Italian context; generalization of our results to other countries would require a much broader scope and more in-depth investigation.

## Implications for Further Research

The results obtained in this study open the way for future research to explore in greater detail the strategies implemented and to clarify the long-term impact of the COVID-19 pandemic on SiHo. To this end, the views of others involved in hospital distance education, especially students and their families, should be explored in future work. In addition, the attitude of healthcare professionals towards the SiHo service should be investigated to develop effective institutional strategies that enhance the professional standing of hospital teachers and, ultimately, their educational effectiveness.

## Additional File

The additional file for this article can be found as follows:

10.5334/cie.26.s1 Appendix. Qualitative data related to the interviews.
