# Supplementary material for: Exploring the Impact of the COVID-19 Pandemic on Italy’s School-in-Hospital (SiHo) Services: The Teachers’ Perspective
Source: Contin Educ. 2020 Dec 11;1(1):136–49. doi: 10.5334/cie.26 (PMC11104330; doi:10.5334/cie.26)
Supplement: Appendix. — Qualitative data related to the interviews. [file cie-1-1-26-s1.pdf]

Appendix

Table 1

Themes and Codes Resulting From the Qualitative Analyses

Themes	Codes	Research Question
**Critical issues in starting distance learning**		RQ1
	• Poor communication with healthcare personnel<br>• Short length of hospital stay<br>• Reorganization of hospital spaces for emergencies<br>• Absence of recognition of the role of the teacher by healthcare personnel<br>• Bureaucracy/privacy that prevents information sharing<br>• Difficulties in initiating contacts with newly hospitalized students<br>• Teaching activities slowed down or prevented<br>•No access to the daily list of hospitalized students	
**Factors Favorable to starting distance learning**		RQ1
	• Fruitful communication with healthcare personnel<br>• Pre-existing contacts with students<br>• Access to the daily list of hospitalized students<br>• Age of students – older students<br>• Length of hospitalization – long stays<br>• Collaboration with colleagues<br>• Flexibility of the hospital teacher's work<br>•Positive response from families<br>• Collaboration with families<br>• Informal relationships with healthcare personnel	
**Strategies used to start distance activities with hospitalized students**		RQ1

	• Posters<br>• Social media<br>• Maintaining contact with colleagues<br>• Maintaining contact with healthcare personnel<br>• Direct contact with the families of hospitalized students<br>• Direct contact with hospitalized students	
**Distance learning with mainstream schools**		RQ1
	• Opportunity to start distance learning with classmates<br>• Easy start to distance learning with classmates<br>• Critical issues – leveling (of curricula)<br>• Critical issues – non-compatible timetables<br>• Critical issues – international students<br>• Critical issues – poor relationships with classmates<br>• Critical issues – excessive workload<br>• Critical issues – health condition of hospitalized students<br>• Hospital school continuous support to students' distance learning with classmates<br>• Initial enthusiasm of hospitalized students for distance learning with classmates<br>• Return to home school distance learning mediated by hospital school<br>• Distance learning not inclusive	
**Methodologies and implementation strategies**		RQ1<br>RQ2
	• Methodological complexity<br>• Use of the same methodologies for face-to-face teaching<br>• Method used: Asynchronous activity<br>• Method used: Short-term teaching activity<br>• Method used: 1: 1 teaching activities<br>• Method used: Individualized teaching<br>• Method used: Didactic recovery activities<br>• Method used: Teamwork	

	• Method used: Mixed course<br>• Overcoming transmissive methodology<br>• Scheduling of activities	
**Tools to support distance learning**		RQ1<br>RQ2
	• Facebook<br>• Synchronous communication tools<br>• Repositories<br>• Video lessons<br>• Helpdesks<br>• Flexibility in the choice of tools<br>• Tools capable of facilitating continuity in the teaching process<br>• Adoption of tools already used by students	
**Critical issues in implementing hospital distance learning**		RQ2
	• Difficulty in contacting newly admitted students<br>• Language issues for international students<br>• Teachers' technological competency<br>• Relational aspects<br>• No core subjects<br>•Families' low skill levels in the use of technology<br>• Students' low skill levels in the use of technology<br>•Need to access the hospital in person<br>• Need for face-to-face contact<br>• Effort required in carrying out distance learning<br>• Need for younger students to be supported by parents	
**Relationships with mainstream schools**		RQ2
	• Need to initiate contact as soon as possible<br>• Importance of maintaining social contact with classmates<br>• Importance of collaboration with mainstream teachers<br>• Creation of hybrid learning spaces (hospital/mainstream school)	
**Need for institutional support**		RQ2
	• Need for support of school manager<br>• Need for national guidelines<br>• Need to disseminate information on the hospital school service<br>• Need to enhance the professional	

	role of the hospital teacher<br>• Need to have a reference person in the healthcare team<br>• Need to involve teachers in the healthcare team	
**Teachers' attitudes towards distance learning and the emergency**		
	• Early reaction – new experience<br>• Early reaction – disorientation<br>• Early reaction – critical phase<br>• Gratitude for sharing<br>• Teacher availability for students' needs<br>• Need to re-think future work<br>• Positive attitude towards the use of technology<br>• Perception of differences in the sections of Italian hospital schools<br>• Positive attitude towards distance learning<br>• Negative attitude towards distance learning<br>• Desire for "normality"	
**Miscellaneous**		
	• Start of new didactic paths during the emergency<br>• Support of experts in distance learning (CLIPSO staff)<br>• Distance learning not inclusive<br>• Feeling of continuity<br>• "External" enhancement of the hospital teacher's skills<br>• Organization of the hospital school before the emergency<br>• Working mobile phone<br>• Training needs<br>• Long-term hospital patients as beneficiaries of hospital school's distance learning<br>• Short-term hospital patients as beneficiaries of mainstream school's distance learning	
